# Single-cell analysis of chromatin and expression reveals age- and sex-associated alterations in the human heart

**DOI:** 10.1038/s42003-024-06582-y

**Published:** 2024-08-26

**Authors:** David F. Read, Gregory T. Booth, Riza M. Daza, Dana L. Jackson, Rula Green Gladden, Sanjay R. Srivatsan, Brent Ewing, Jennifer M. Franks, Cailyn H. Spurrell, Anne Roshella Gomes, Diana O’Day, Aishwarya A. Gogate, Beth K. Martin, Haleigh Larson, Christian Pfleger, Lea Starita, Yiing Lin, Jay Shendure, Shin Lin, Cole Trapnell

**Affiliations:** 1 Department of Genome Sciences, University of Washington, Seattle, WA USA; 2 Brotman Baty Institute for Precision Medicine, Seattle, WA USA; 3 Seattle Children’s Research Institute, Seattle, WA USA; 4 University of Washington School of Medicine, Division of Cardiology, Seattle, WA USA; 5 Department of Surgery, Washington University, St Louis, MO USA; 6 Howard Hughes Medical Institute, Seattle, WA USA; 7 Allen Discovery Center for Cell Lineage Tracing, Seattle, WA USA

**Keywords:** Cardiovascular biology, Gene expression, Gene regulation

## Abstract

Sex differences and age-related changes in the human heart at the tissue, cell, and molecular level have been well-documented and many may be relevant for cardiovascular disease. However, how molecular programs within individual cell types vary across individuals by age and sex remains poorly characterized. To better understand this variation, we performed single-nucleus combinatorial indexing (sci) ATAC- and RNA-Seq in human heart samples from nine donors. We identify hundreds of differentially expressed genes by age and sex and find epigenetic signatures of variation in ATAC-Seq data in this discovery cohort. We then scale up our single-cell RNA-Seq analysis by combining our data with five recently published single nucleus RNA-Seq datasets of healthy adult hearts. We find variation such as metabolic alterations by sex and immune changes by age in differential expression tests, as well as alterations in abundance of cardiomyocytes by sex and neurons with age. In addition, we compare our adult-derived ATAC-Seq profiles to analogous fetal cell types to identify putative developmental-stage-specific regulatory factors. Finally, we train predictive models of cell-type-specific RNA expression levels utilizing ATAC-Seq profiles to link distal regulatory sequences to promoters, quantifying the predictive value of a simple TF-to-expression regulatory grammar and identifying cell-type-specific TFs. Our analysis represents the largest single-cell analysis of cardiac variation by age and sex to date and provides a resource for further study of healthy cardiac variation and transcriptional regulation at single-cell resolution.

## Abstract (web summary)

Analysis of single-nucleus RNA- and ATAC-Seq from healthy human heart revealed age- and sex-specific alterations, defines candidate developmental stage-specific regulatory patterns, and identifies cell type-specific regulatory signatures.

## Introduction

Profound alterations in cardiac function and disease risk have long been evident at the level of individuals’ traits such as sex^1^ and age^2^. For example, female hearts exhibit more modest declines in cardiomyocyte numbers over time than males^3^ and display distinct vascular elasticity properties^4^, while aged hearts display ventricular hypertrophy, tissue stiffening, and inflammation^2,5^. However, there is substantial uncertainty in the exact molecular and cellular hallmarks - much less causal mechanisms - of these clinically evident, consequential differences. A robust understanding of those molecular processes could set the stage for personalized therapeutic intervention.

To achieve cell-type-resolved but high-throughput measurements of cardiac biology, single-cell methods have been employed in numerous studies of human hearts and model organisms. In humans, these analyses profiled the diversity of cardiac cell types and subtypes^6,7^ and generated genome-wide maps of cell-type-specific regulatory programs^8^. In model organisms, single-cell approaches have not only generated atlases of healthy tissue^9,10^ but have also been used in controlled experiments to dissect the alterations occurring in processes such as aging^11^ and heart disease^12–14^. Similar approaches have begun to profile clinically important contrasts in human samples directly, such as identifying a handful of transcripts that vary by age in the healthy human heart^7^, variation in myeloid cell abundance in age^15^, and alterations during disease in single-cell ATAC-Seq^8^ and RNA-Seq^15^ data. Further analyses and larger datasets of the human heart may unlock more extensive insights into how alterations in transcriptional and epigenetic states characterize variation between individuals and advance our understanding of the genomic programs regulating cells.

Chromatin regulation represents a significant element of specialized cell function within or between conditions. During development, transcription factors play variable roles over the course of cardiac development^16,17^. Of clinical concern, individual transcription factors may play decisive roles in diseases such as cardiac fibrosis^18^ while genetic variation may act through regulatory mechanisms to affect disease risk and individual variation^19^. Parallel advances in quantitative models of gene expression^20–22^ and extensive generation of epigenetic datasets in primary human hearts^8,17,23^ bode well for the utility of further, diverse epigenetic datasets in revealing intra- and inter-state regulatory programs.

To extend knowledge of molecular cell-type-specific cardiac processes between and within individuals, we generated and analyzed matched single-cell ATAC-Seq (117,738 cells) and RNA-Seq (89,404 cells) datasets from 15 samples spanning 9 individuals. As a resource, our dataset contributes substantially to the catalog of single-cell profiles of the human heart. The number of individuals profiled combined with a hierarchical mixed model regression approach allows us to resolve age- and sex-dependent transcriptional and chromatin accessibility changes apart from confounding by donor-level variation. We find that transcriptional and regulatory programs display widespread variation by these covariates, observing both cell-type-specific and largely pan-cell-type alterations. For example, sex was associated with alterations in transcriptional signatures of oxidative phosphorylation as well as differing accessibility at ATAC-Seq peaks containing motifs of TFs known to regulate metabolic rewiring. Motivated by these differences in our newly generated data, we combined our snRNA-Seq data with five additional datasets of snRNA-Seq and ran a meta-analysis of variation by age and sex at the level of transcription and cell-type composition. Furthermore, as one of a small number of resources of single-cell-resolved ATAC-Seq information in human heart we utilized ATAC-Seq information to interrogate the regulatory grammar of the human heart in two ways. First, we utilize newly generated ATAC-Seq data to identify putative life-stage-specific TFs, finding indications of adult-specific activity by RFX family TFs in adult vascular endothelium and macrophages. Second, we develop cell-type-specific gene expression models that utilize informative distal regulatory sites to account for approximately a quarter of transcriptional variation using a simple TF motif regulatory code and define quantitative roles of regulatory motifs by cell type.

## Results

### Single-cell ATAC- and RNA-Seq library generation and cell annotation

**Fig. 1:**
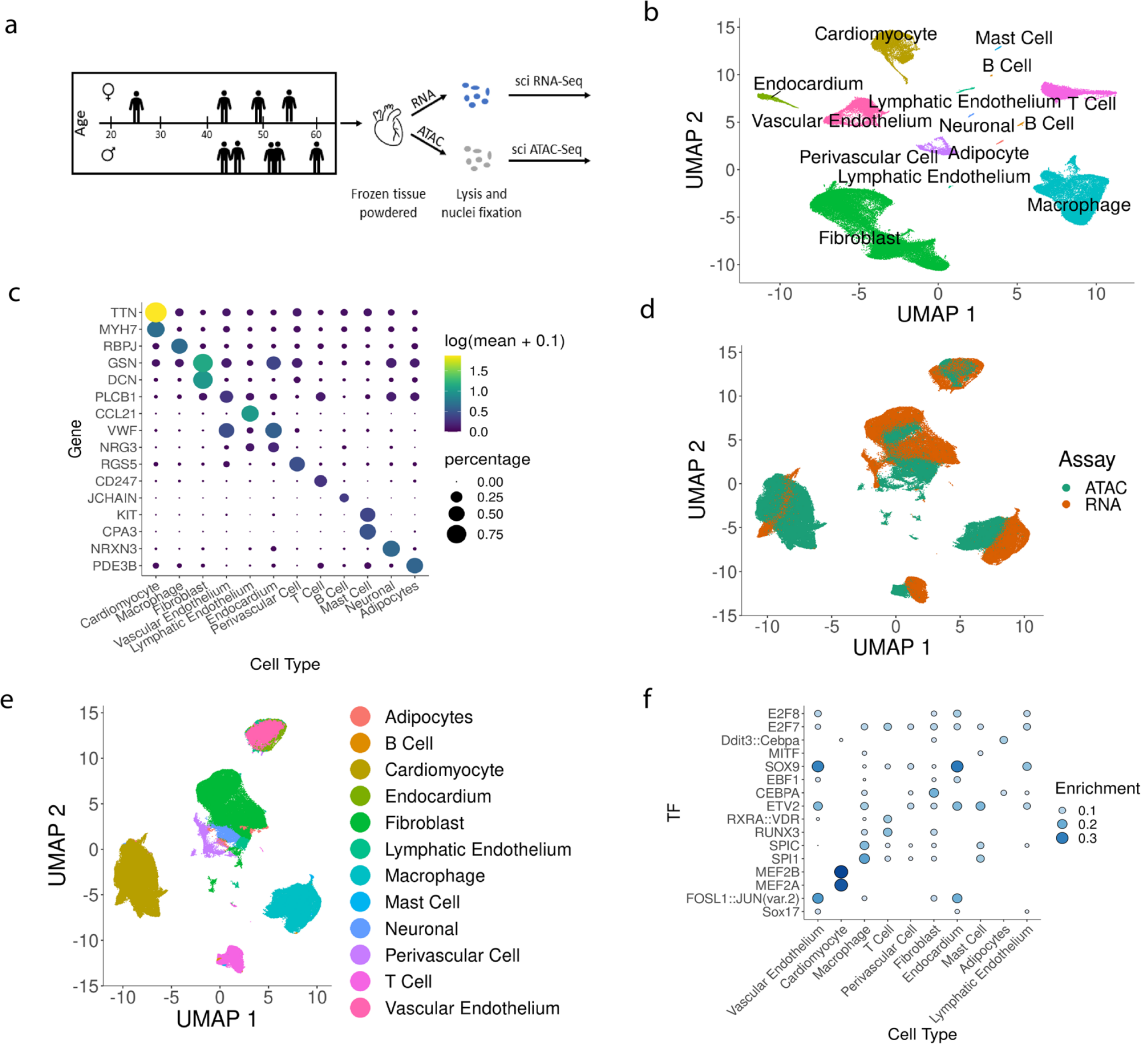
Overview of datasets. **a** Experimental setup. **b** UMAP of RNA-Seq data, coloring be cell type. **c** Plot of marker gene expression by cell type, showing both mean expression (dot color) and the proportion of each cell type with nonzero expression for that marker (dot size). **d** UMAP of a co-embedding of snRNA-and ATAC-Seq data, coloring by data type. **e** UMAP of co-embedded RNA and ATAC data, coloring by the assigned cell type. **f** Enrichment of TF motifis in the accessible peaks of ATAC-Seq cells.

We collected heart samples from nine healthy adult donors (Supplementary Data 1) in a manner that minimized warm ischemic time. Donors were included based on clinician assessment of health and ability for samples to be collected under standardized protocols (see “Methods” section). Samples represented four anatomical sites from the heart, though almost all were collected from the heart apex or left ventricular wall and no samples were from heart atria (Supplementary Data 1). In total, we prepared 15 samples from 9 donors for single-cell analysis, with each sample representing a single anatomical site in a particular donor. We powdered frozen tissue and split portions into aliquots for appropriate nuclei isolation and fixation for ATAC-Seq and RNA-Seq separately (Fig. 1a).

We generated single-nuclei RNA-Seq libraries using 3-level sci RNA-Seq^24^. We modified the nuclei isolation protocol to use additional RNase inhibitors, mechanical dissociation of tissue, and 5% glutaraldehyde for tissue fixation (see “Methods” section). Additionally, in order to reduce background RNA levels that commonly contribute noise to single-cell RNA-Seq data^25^ we included a FACS sorting step following ligation. One sample failed completely (donor “W137”) but the remaining 14 samples – spanning 8 unique donors - yielded 89,404 nuclei after doublet removal and filtering. A UMAP embedding of all transcriptomes contained numerous clearly separated clusters, with contributions from distinct samples across clusters (Supplementary Fig. 1A).

Examination of marker genes revealed that clusters corresponded to specialized cells of the human heart including cardiomyocytes, fibroblasts, macrophages, and endothelial cells (Fig. 1b, c). The RNA data also clearly resolves rare cell populations such as adipocytes, neuronal cells, mast cells, and B cells (Fig. 1b).

Separately, we prepared single-nuclei ATAC-Seq data from powdered frozen tissue using 3-level sci ATAC-Seq^23^, covering 15 samples across 9 donors and generating 117,738 ATAC-Seq profiles after filtering and doublet removal. Single-cell ATAC-Seq data is more difficult to annotate than RNA data because open chromatin around a gene doesn’t always indicate that the gene is robustly expressed^26^. Due to this difficulty in defining cell types, we used a co-embedding approach to find a low-dimensional embedding of RNA- and ATAC-Seq data simultaneously (Fig. 1d), then transferred cell-type labels from RNA to ATAC profiles using a k-nearest neighbor classifier (Fig. 1e; see “Methods” section; see Supplementary Fig. 2 for post-filtering QC). Using these assignments, within the ATAC-Seq data we identify strong enrichments for expected cell-type-specific transcription factors (Fig. 1f) such as MEF2 family transcription factors in cardiomyocytes, SPI1 (also known as PU.1) in macrophages, and CEBPA in fibroblasts, in agreement with recent analyses of adult single-cell ATAC-Seq data in adult human hearts^8^.

### Sex- and age-dependent variation is evident in transcriptional and epigenetic data in a preliminary analysis

**Fig. 2:**
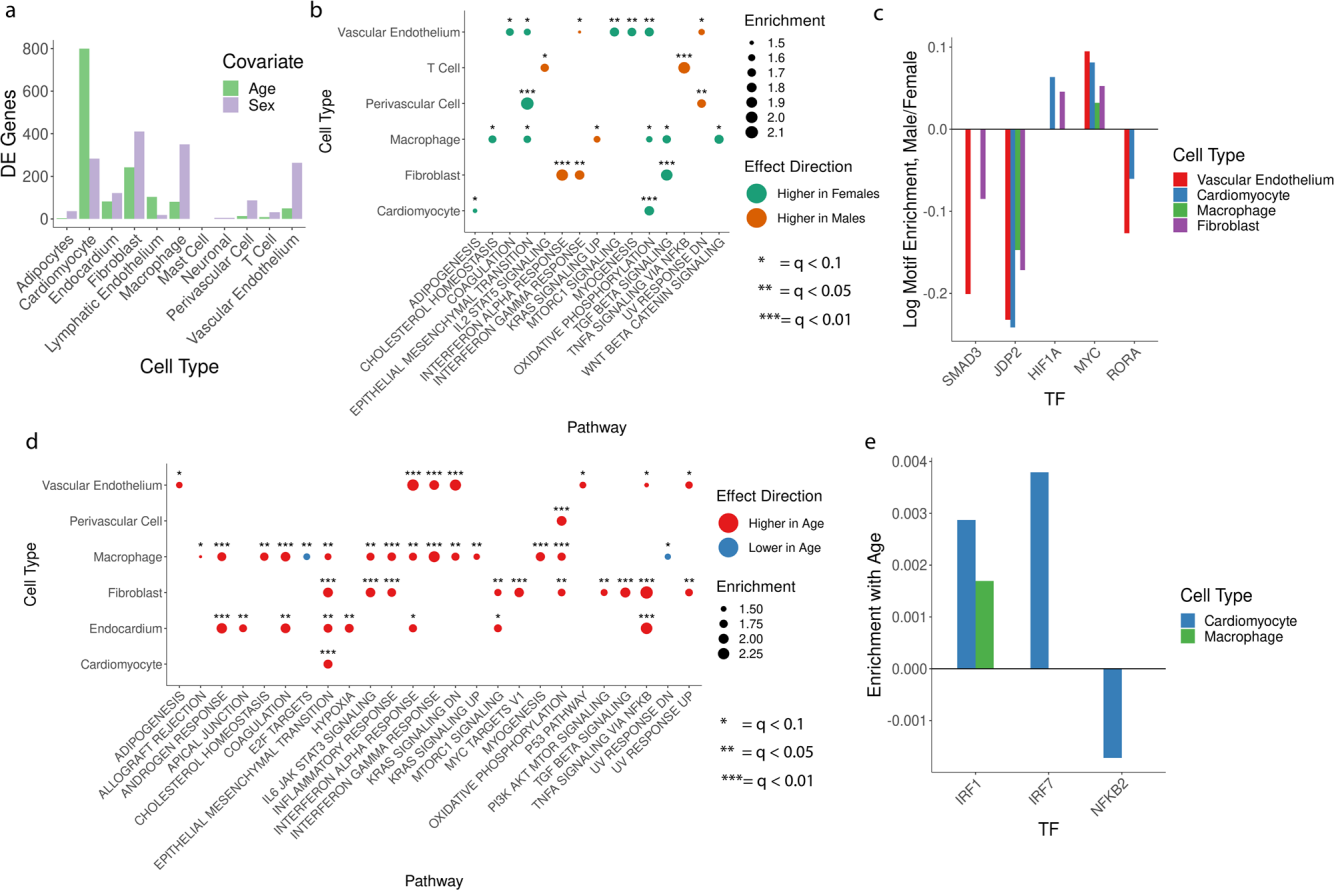
Sex- and age-associated gene expression changes in the heart. **a** Differentially expressed genes as a function of age (green) or sex (purple), FDR- 0.1. Testing used a mixed effect model to control for variation by anatomical site, cell UMI, and inter-donor variation, multiple-testing corrected using the Benjamini–Hochberg method. Total donors *n* = 8. **b** G5EA by biological pathways as a function of sex. Greer = pathway components up-regulated in females, orange = up-regulated in males. Total donors *n* = 8. Asterisk number indicates the FDR threshold of significance. **c** TF motifs enriched or depleted in accessible chromatin as a function of sexus-ing a mixed effect model controlling for age, sex, anatomical site, read depth, and inter-donor variation (see “Methods” section), FDR = 0.1. Total donors *n* = 9, **d** GSFA by biological pathways as a function of sex. Red = pathway up-regulated with age, blue = down-regulated with age. Unique donors *n* = 8. **e** TF motifs enriched or depleted in accessible chromatin as a function of age, using the same approach as (**c**).

We explored our single-cell RNA-Seq data to identify transcriptional changes associated with age or sex. Commonly used single-cell differential expression methods using fixed effect regression models do not properly account for inter-donor variation and can dramatically inflate false discoveries^27,28^. Consequently, we used a mixed effect modeling framework to test for differential expression (see “Methods” section) that allowed us to test for variation by sex and age while controlling for expression differences due to anatomical site and read depth. We find dozens to hundreds of differentially expressed genes by age and sex, depending on the cell type analyzed (Fig. 2a). Most DE genes are found in relatively abundant cell types, and we note particularly large numbers of changes by age in cardiomyocytes as well as many differences by sex in fibroblasts, macrophages, and vascular endothelium.

To summarize transcriptional sex- and age-specific variation, we tested for enrichment in up- and down-regulated gene sets. Statistically significant differences between male and female hearts were evident across several cell types (Fig. 2a, Supplementary Data 2 and 3). We observe decreased expression of target genes of TGFB signaling in male fibroblasts and macrophages as well as decreased hallmarks of epithelial-to-mesenchymal transition (EMT) - a common downstream consequence of TGFB activity ^29^ - in macrophages, vascular endothelial, and perivascular cells (Fig. 2b). Additionally, we find statistically significant changes in genes important in various aspects of cell metabolism as a function of sex, including decreased expression of cholesterol metabolism-associated transcripts in macrophages and decreased oxidative phosphorylation-related transcripts in male cardiomyocytes, vascular endothelium, and macrophages (Fig. 2b). We also observe cell-type-specific alterations, such as an increase in hallmarks of IL2 and TNFA signaling in male T cells (Fig. 2b) consistent with elevated soluble inflammatory signaling^30,31^.

To study regulatory programs whose activity differed by sex, we looked for alterations in TF motifs accessibility between male and female cells in our sn ATAC-Seq (Supplementary Data 4**)**. Consistent with altered TGFB signaling in transcriptional data (Fig. 2b), we see reduced abundance of motifs for SMAD3 – a downstream effector of canonical TGFβ signaling – in male fibroblasts and vascular endothelial cells (Fig. 2c). We further see statistically significant decreases in accessible motifs corresponding to JDP2, a transcriptional repressor tied to alteration of TGFB1-induced EMT and fibrosis^32,33^. Consistent with decreased expression of hallmarks of oxidative phosphorylation in males (Fig. 2b), we detect sex-specific changes across multiple cell types in HIF1A, MYC, and RORA, TFs known to promote glycolysis over oxidative phosphorylation^34^. We do note discrepancies between ATAC-Seq- and RNA-Seq-based in the exact cell types in which alterations were observed. For instance, while there was variation in TGFβ signaling hallmarks in transcriptional data from macrophages (Fig. 2b) we did not detect variation in SMAD3 motifs by sex macrophages (Fig. 2c). Similarly, cardiomyocytes display altered JDP2 motif abundance by sex (Fig. 2c) but no changes in TGFβ or EMT pathways are detected in cardiomyocytes transcriptionally (Fig. 2b). However, imperfect alignment in changes detected via ATAC-Seq and RNA-Seq analyses is unsurprising (see “Discussion” section) and our results highlight potential TF mediators underlying transcriptionally detected alterations.

We next explored cell-type-specific changes in the expression of hundreds of genes that varied by donor age (Fig. 2a). We again tested for enrichments in gene sets for age-related expression changes within individual cell types. We found a variety of alterations, including changes in several metabolic and cell-signaling pathways (Fig. 2d). We again observed differences in TGFB signaling genes, with increased TGFB hallmarks increasing in aged fibroblasts and epithelial-to-mesenchymal transition-associated transcripts elevated in aged fibroblasts, macrophages, endocardial cells, and cardiomyocytes (Fig. 2d**)**. Age is associated with an increase in several immune pathways across several cell types, including hallmarks of inflammation in fibroblasts and macrophages and increased interferon response in macrophages and vascular endothelium (Fig. 2d).

To see if age-dependent alterations in immune activation hallmarks were evident at the level of chromatin remodeling, we tested for motif enrichment in the accessible peaks as a function of age (Supplementary Data 5). We detected enrichment of IRF1 and IRF7 motifs in the accessible peaks of cardiomyocytes, fibroblasts, macrophages, and vascular endothelial cells (Fig. 2e) consistent with increases in interferon response pathways observed in our transcriptional data (Fig. 2d). In addition, we observe a statistically significant decrease in accessible motifs for NFKB2 (Fig. 2e), a central mediator of inflammatory signaling^35^. These changes in the accessibility of motifs corresponding to key mediators of immune activation identify potential regulatory changes accompanying or underlying observed transcriptional changes (Fig. 2d). Still, as was observed in the analysis of sex-dependent changes in motif abundances (Fig. 2c) we note subtle distinctions between cell types in which transcriptional and epigenetic changes are detected. For instance, while we saw enrichment of IRF1 and IRF7 motifs in cardiomyocytes, fibroblasts, macrophages, and vascular endothelium (Fig. 2e) we note that transcriptional changes in interferon response hallmarks were observed only in macrophages, vascular endothelium, and endocardium (Fig. 2d, see “Discussion” section).

In addition, we examined variation in cell-type proportions as a function of sex and age. Broadly, cell-type proportions were consistent between the sexes. However, we observe some differences in cell proportions by age (Supplementary Data 6) including altered neuronal, vascular endothelial, and perivascular proportions.

### Meta-analysis of sex- and age-dependent variation identifies altered cell-type proportions and expression

**Fig. 3:**
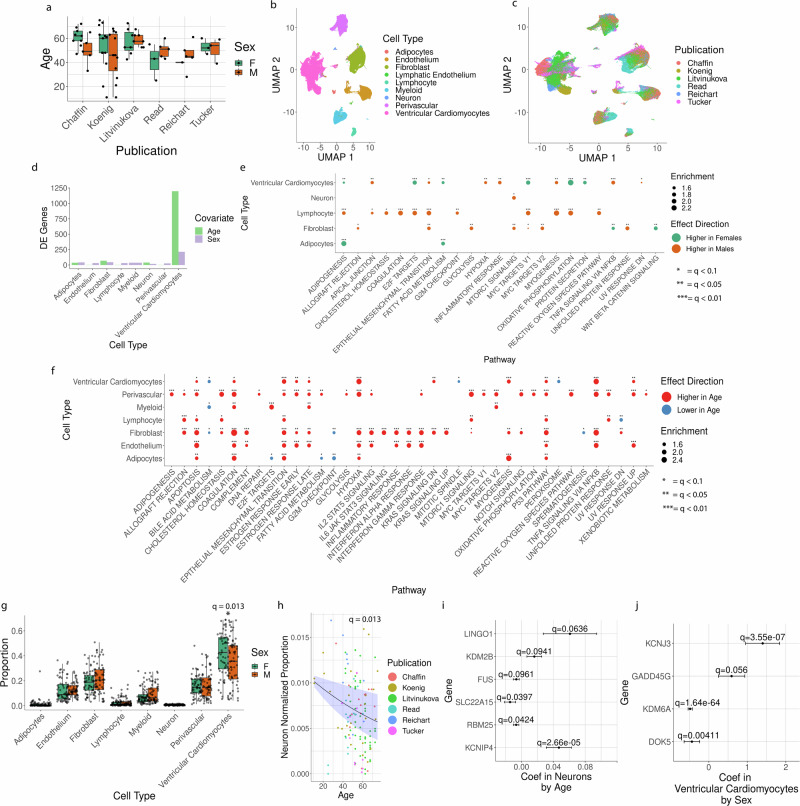
Meta-analysis of sex- and age-associated expression patterns across studies. **a** Age and sex of all unique donors (one point = one donor) by publication. **b** UMAP embedding of single-nuclei RNA-Seq data from six studies, colored by cell type. **c** UMAP embedding as in (**b**), coloring points (cells) by data source. **d** Number of differentially expressed genes as a function of age (green) or sex (purple), FDR = 0.1. Testing used a mixed effect model controlling for variation by anatomical site, cell UMI, data source, and inter-donor variation (see “Methods” section). Uses *n* = 73 donors. **e** GSEA by biological pathways as a function of sex, looking at sex-dependent variation as fit using a mixed effect model (see “Methods” section). Green = pathway components up-regulated in females, orange = up-regulated in males. Total donors *n* = 73. Asterisk number indicates FDR threshold of significance. Benjamini–Hochberg method for multiple-testing correction. **f** GSEA by biological pathways as a function of sex. Red = pathway up-regulated with age, blue = down-regulated with age. Unique donors *n* = 73. **g** Plot of cell-type proportions per sample by sex. Note that one point represents one technical sample, *n* = 73 unique donors analyzed. **h** Plot of the adjusted proportion of neurons in a given sample by age, colored by data source. Note that *n* = 73 unique donors, while in this plot one point = one biological sample (repeated samples from distinct sites). Trend line represents the predicted proportion of neurons from a beta-binomial mixed effect model (see “Methods” section) with the uncertainty range representing predictions using an age coefficient ± 2 standard errors around the fit value for the age effect. Proportions were adjusted to remove effects by sex, anatomical site, and data source based on coefficients fit for those variables (see “Methods” section). **i** Coefficients genes as a function of age in a mixed effect model fit in neurons. Error bars represent coefficient estimate ± 2 standard errors. *Q*-values listed are calculated from *p*-values using the Benjamini–Hochberg method. **j** Coefficients for genes as a function of sex (positive coefficient = higher expression in males, negative = higher expression in females) in a mixed effect model fit in cardiomyocytes. Error bars represent coefficient estimate ± 2 standard errors as in (**i**).

Our parallel generation of multiple data modalities allowed us to study variation in cell-type proportions and RNA expression (via snRNA-Seq) and epigenetic state (via snATAC-Seq) as a function of age and sex. Given the scale of inter-donor variability and our newly generated data spanning only nine unique individuals, more data was desirable. However, due to limited access to suitable donor samples as well as the cost and complexity of single-nucleus RNA- and ATAC-Seq data generation, drastically increasing the number of profiled donors ourselves was infeasible. To increase our

sample size, in our next step we combined five additional datasets of snRNA-Seq data^6,7,15,36,37^ with our own for a total of 73 unique donors worth of snRNA-Seq data from adult, non-failing human hearts (Fig. 3a, see “Methods” section). Given the limited number of donors with any available data from atrial samples, we restricted our analysis to samples from non-atrial sites.

After combining all six datasets, we qualitatively checked for any obvious discrepancies in cell-type annotations or drastic inter-source effects. In a UMAP embedding generated from all six studies’ worth of single-nucleus RNA-Seq data, after correcting for data source using mutual nearest neighbors (see “Methods” section) we saw good agreement between cell-type assignments in different studies. After harmonizing naming conventions (Supplementary Data 7), cells with the same annotation clustered together (Fig. 3b) with contributions from each study across clusters (Fig. 3c). Given this, we did not re-assign cell types but instead kept labels as set by respective studies. We also concluded that while inter-study effects needed to be accounted for, they did not appear to be so drastic as to prevent the inclusion of any study from a meta-analysis. Transcriptional variation by the anatomical site is evident (Supplementary Fig. 3, Supplementary Data 8–10), but given extensive interrogation of variation by the anatomical site in two of our included datasets^6,7^ we focused our analysis on variation by age and sex. After these initial data quality checks, we repeated our analyses of differential expression and differential cell-type proportions as a function of age and sex in the combined dataset.

We tested for differential expression as a function of age or sex while controlling for variation by study, donor, read depth, and anatomical site using a mixed model framework (see “Methods” section). In contrast to widespread differential expression across cell types in our initial analysis of our dataset alone (Fig. 2a), we find that differentially expressed genes in the meta-analysis were strongly concentrated in cardiomyocytes with few statistically significant alterations detected in other cell types (Fig. 3d, Supplementary Data 11 and 12). However, statistically significant alterations at the level of biological pathways were spread across many cell types as a function of sex (Fig. 3e) and age (Fig. 3f). At the level of pathway-wide changes, many changes seen in the analysis of the smaller cohort (Fig. 2b, d) were reproduced in the larger analysis. For example, we find alterations in metabolic pathways as a function of sex (Fig. 3e) as well as widespread changes in immune-related pathways, lipid metabolism, and UV-response pathways as a function of age (Fig. 3f). However, we additionally see cases where results are not replicated, such as not reproducing increased TGFβ signaling-related transcription in females (Fig. 3e) as was observed in the analysis of newly generated data alone (Fig. 2b). The observation of some such differences is unsurprising - given the likely complex underlying biology, inherent variation between individuals, and lack of detailed information on confounders - and highlights the value of significantly scaling up our analyses through incorporation of additional datasets (see “Discussion” section).

To test for alterations in the cell-type composition of the heart, we used a beta-binomial mixed effect model to test for alterations in proportions of individual cell types as a function of age or sex, while simultaneously controlling for study, donor, and anatomical site (see “Methods” section). We find a statistically significant difference in cardiomyocyte proportions as a function of sex (Fig. 3g, *q* = 0.013), consistent with previous reports of altered cardiomyocyte proportions between male and female hearts^3^. In addition, we see statistically significant decreases in neurons (Fig. 3h, *q* = 0.013) as a function of age, as well as changes in adipocytes and myeloid cells (Supplementary Data 13, *q* = 0.094 and 0.098, respectively).

Motivated by our observation of altered neuron and cardiomyocyte proportions as a function of age and sex, respectively, we examined the list of DE genes for those cell types as a function of those variables. In neurons, we note age-varying expression in LINGO1^38,39^, KDM2B^40^, and FUS^41^, genes linked to neuron or neural progenitor survival and regeneration (Fig. 3i). We also see age-associated variation in genes associated with solute transport (SLC22A15^42^ and RBM25^43^) and neuron excitability (KCNIP4^44^) (Fig. 3i). In cardiomyocytes, we see sex-related variation in genes related to cardiovascular disease and cardiomyocyte preservation, such as KCNJ3 (higher in males, associated with arrythmias^45^), GADD5G (higher in males, promotes cardiomyocyte apoptosis in heart failure^46^), KDM6A (higher in females, protects cardiomyocytes from hypoxia-induced apoptosis^47^), and DOK5 (higher in females, a driver in cardiomyocyte differentiation^48^).

### Contrasting TF motif enrichments identify putative adult- and fetal-specific regulators

To study the global relationship between TF activity in adult cells versus their embryonic counterparts, we used a regression approach to identify TF motifs enriched in accessible chromatin of specific cell types (see “Methods” section) and compared the enriched motifs in adult cell types against corresponding fetal cell types^23^. Motif accessibility in most fetal cell types was largely maintained in the corresponding adult types (Supplementary Data 14), though concordance was weaker for less abundant cell types such as adipocytes, or cell types for which our matching approach was less confident (e.g., fetal perivascular cells and adult smooth muscle cells) (Supplementary Fig. 4).

**Fig. 4:**
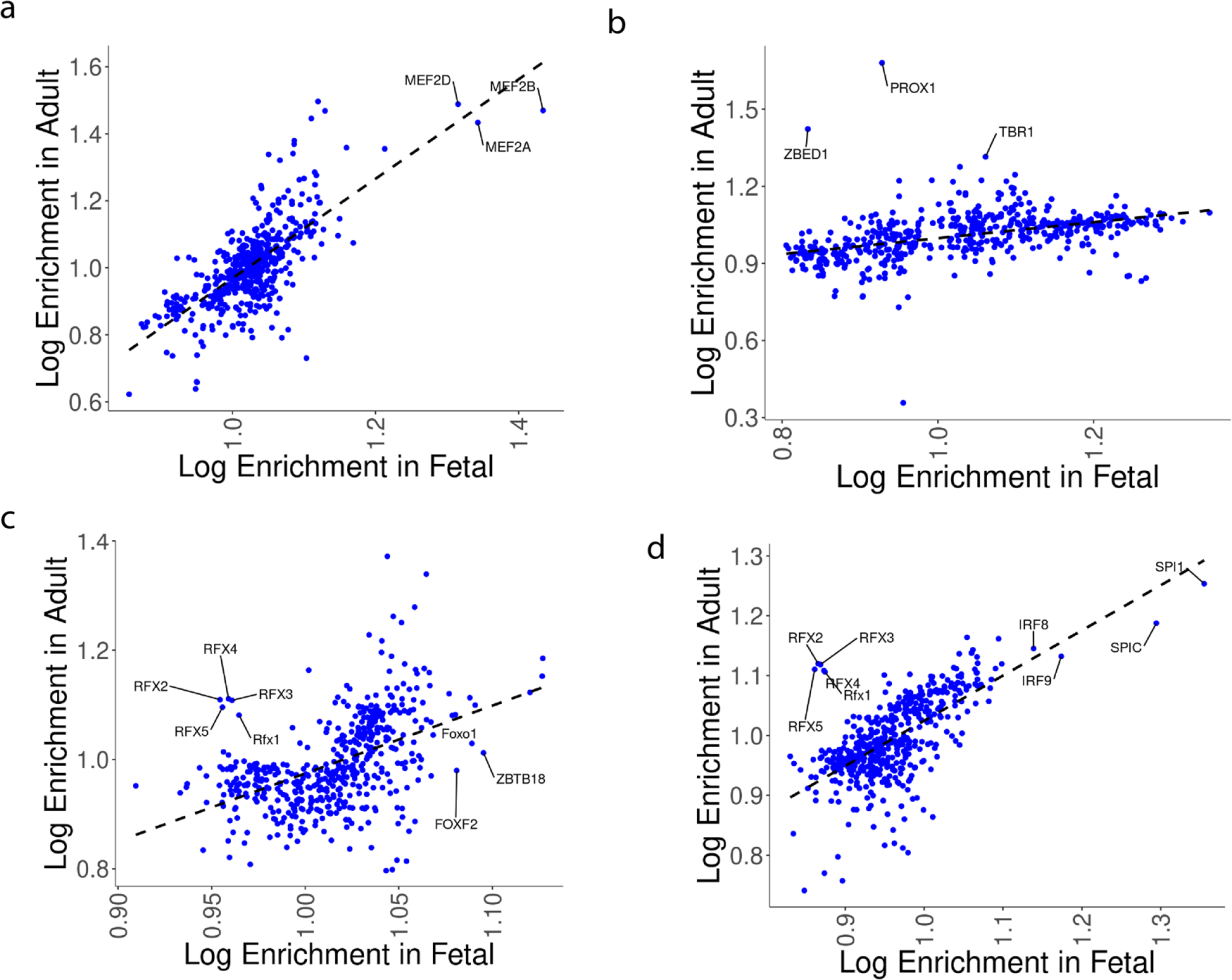
Enrichment of TF motifs in the accessible peaks of fetal or adult sn ATAC-Seq. **a** Enrichments in cardiomyocytes. **b** Enrichments in cardiac neurons. **c** Enrichments in vascular endothelial cells. Enrichments are shown for TFs that were statistically enriched in one or both of fetal or adult analyses (FDR = 0.1). **d** Enrichments in macrophages (adult) versus myeloid lineage cells (fetal).

We next sought adult- or fetal-specific regulatory factors by looking for outliers whose enrichment was markedly higher in one developmental context versus the other. For some cell types, we see few if any obvious discrepancies in TF enrichments between fetal and adult cells. For example, TF enrichments in cardiomyocytes are highly correlated between our analysis and corresponding results from fetal data including MEF2 regulators being exceptionally enriched in accessible fetal and adult cardiomyocyte chromatin (Fig. 4a). This is consistent with MEF2 TFs playing a crucial role in both cardiomyocyte differentiation and maintenance^49^.

Although the overwhelming majority of TF motifs were similarly enriched in both fetal and adult cells, apparent differences in motif enrichments occur between fetal and adult cell types. For example, while TF enrichment magnitudes are broadly correlated between adult heart and fetal neurons (Pearson correlation = 0.42, *p* = 3.6e−25). Figure 4b, a handful of factors that show higher enrichment in adult neurons. Cardiac neurons play a pivotal role in regulating cardiac electrical and mechanical activity through a combination of intrinsic and central-nervous-system interfacing interactions^50^, while cardiac neuron dysfunction is central in cardiac arrhythmias^51,52^. In adult cardiac neurons we see notable enrichment for PROX1, ZBED1, and TBR1 motifs in accessible chromatin, contrasting with minimal enrichment (or depletion) of those motifs in fetal cardiac neurons (Fig. 4b). PROX1 plays a role in cell cycle exit and terminal differentiation of neurons in the central nervous system^53^ while TBR1 is essential for neural specification in the developing cortex^54^. ZBED1 plays roles in suppressing cell division^55^, but apart from possible interactions between a ZBED1 homolog and a regulator of optic lobe formation in *Drosophila*^55^, ZBED1 has not been previously characterized as a neural regulatory factor. In contrast to adult-specific motif enrichments, we observe fetal-specific enrichments of factors such as NOTO, a regulator of notochord lineage commitment^56^, and RORA, a regulator of CNS development^57^. Altogether, our results show that while some factors are shared between fetal and adult cardiac neurons, others may be developmentally specific.

In fetal vascular endothelial cells, we see enrichment specifically in fetal cells for known vasculature regulators FOXO1 and FOXF, both of which cause severe vascular remodeling defects and embryonic lethality upon knockout in mice^58^ (Fig. 4c). In addition, we see a similar level of enrichment in accessible fetal chromatin for ZBTB18 in contrast to minimal enrichment in adult vascular endothelium (Fig. 4c). In the opposite direction, we see adult-specific enrichment for five RFX factor motifs (Fig. 4c) in adult vascular endothelium. Interestingly, we see adult-specific enrichment for these motifs in a comparison of adult versus fetal macrophages as well (Fig. 4d). Both of these cell types play crucial roles in vascular dysfunction^59^, while RFX factors are correlated with epigenetic changes in hypertension patients^60^ and RFX1 indirectly reduces monocyte recruitment in atherosclerosis^61^. Given motif similarities between RFX factors, further work will be particularly important to understand the role of RFX TFs in endothelial and macrophage function. For now, our work raises the potential for adult-specific roles for RFX factors in cardiac endothelium and macrophages joining RFX factors’ previously characterized pleiotropic roles^62^.

Overall, our comparison of cell-type-specific fetal and adult motif enrichments tells us two things. First, we reproduce the observed correlation across various tissues between corresponding adult and fetal cell types’ chromatin in terms of accessible motifs^17^. Second, motifs that are not correlated between fetal and adult cells identify candidates for developmental-stage-specific regulators in cardiac cell types.

### ATAC-Seq links distal sites that improve predictive models of RNA expression

Given the small number and recent publication of datasets studying the adult human heart with single-cell resolved ATAC-Seq data^8,17^ we additionally utilized the single-nucleus ATAC-Seq data to develop predictive models of gene expression. We aimed to fit simple, interpretable models that could provide insights into the role of regulatory factors in individual cell types.

Characterizing the regulatory roles of noncoding DNA sequences is a pressing challenge in human genetics^63^. Although a handful of distal elements with significant functional roles in the heart have been characterized^18^ and genome-wide maps of cis-regulatory elements have recently been published^8^, we lack a genome-scale quantitative model of how noncoding sequences drives gene regulation. One approach to linking sequence to transcription has been to train computational models that predict each gene’s expression based on nearby sequences and/or epigenetic features^64–68^. For example, we previously predicted gene expression based on sequence motifs in the accessible chromatin of differentiating myoblasts and found that simple transcription factor motif presence/absence explained ~37% of transcriptional changes during differentiation^69^. Strikingly, information from distal DNA sequences dramatically improved accuracy compared to a model that used only the promoter sequence, suggesting that much of the information needed to encode the cell-state-specific expression resides in distal sequences^69^. However, the extent to which such models generalize beyond simple in vitro systems to multiple in vivo human cell types is not clear.

**Fig. 5:**
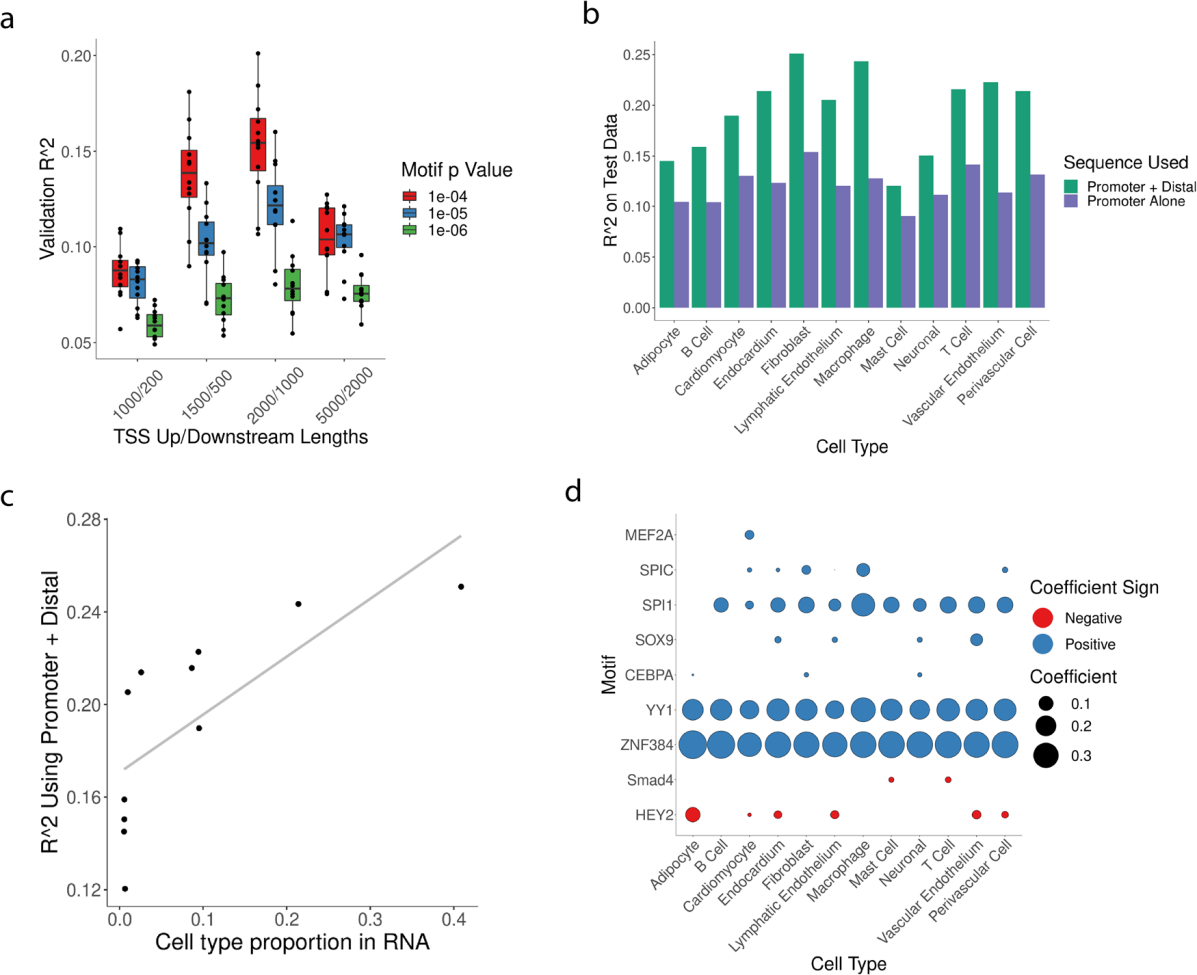
Predictive models of RNA expression. **a** *R*^2^ for cell-type-specific models (one point = one cell type) using varied *p-*value cutoffs for calling motif presence in FIMO (color) and upstream/downstream regions (in bp) with respect to geneTSS. **b** Test set *R*^2^ for different cell types, *R*^2^ is listed for models that were trained using motifs found near geneTSS (in purple) or in either TSS or linked distal sites (in green). **c** Model accuracy on test data for models using promoter and distal motifs (x-axis) and the proportion of that cell type in the snRNA-Seq data (y-axis). **d** Magnitude and direction of motif coefficients in final models fit using distal and proximal sequence.

To assess the potential of each cell type’s accessible chromatin to predict its transcriptome, we modeled cell-type-specific average gene expression based on promoter sequence alone or in combination with distal sites linked by ATAC-Seq information, as in our previous work^69^. We defined hyperparameters for these cell-type-specific expression models using a training set, holding aside two separate validation and test sets to measure model performance. Protein-coding genes were split into train/validation/test sets at the level of whole chromosomes (see “Methods” section) in proportions of approximately 80% train/10% validation/10% test. We found that in models using only promoter sequence the best average predictive accuracy occurred when the promoter region covered 2000 bases upstream and 1000 bases downstream of a TSS (Fig. 5a), while the use of larger or smaller regions led to inferior accuracy. After finalizing all hyperparameters (Supplementary Fig. 5A), two models were fitted for each cell type: One used motifs absence/presence in a promoter region only as input features, while a second model used motifs found in promoters or distal DNA sites. In every cell type, models fit using proximal and distal sequence outperformed the corresponding model using promoter motifs alone, for several cell types by nearly 2-fold (Fig. 5b). Notably, this effect does not appear to be due simply to adding additional arbitrary sequence as use of an even larger promoter region reduced model accuracy (Fig. 5a).

Because models for some cell types were more accurate than others (Fig. 5b), we investigated if model performance was related to the abundance of the cell types. We found that cell-type abundance - as quantified by a type’s proportion of total cells in RNA-Seq data - was related to accuracy for each respective cell type for models trained on promoter sequence alone (Supplementary Fig. 5B) or distal sequence plus promoter sequence (Fig. 5c). Additionally, models for abundant cell types were markedly improved by including distal information, whereas models for less abundant cell types benefitted less (Supplementary Fig. 5C) which suggests that collecting further data would improve our models of gene expression. For abundant cell types, a simple model that predicted each gene’s expression based on whether or not each motif was present in the accessible chromatin nearby was able to account for ~20–25% of expression variation at the level of pseudo-bulked transcriptomes, and that around a third of that amount was due to the inclusion of distal motif information. These results demonstrate that for all cell types of the human heart for which we trained models, distal noncoding DNA improves the accuracy of predicted expression.

We next scrutinized the features used by our models to identify the specific sequences that define cell-type-specific gene expression. As expected, many motifs that predict expression were also detected as enriched in accessible chromatin (Fig. 1g). Examples include motifs for SPIC and SPI1 in the macrophage model, MEF2A in the cardiomyocyte model, SOX9 in the vascular endothelial and endocardium models, and CEBPA in the fibroblast model (Fig. 5d). The models also explicitly identified motifs that are predictive of reduced expression, identifying putative repressors. For example, motifs for HEY2 - which contributes to cardiomyocyte specification in development^70^ - were inversely associated with expression in cardiomyocytes and other cell types (Fig. 4d). This result agrees with the factor’s known role as a transcriptional repressor^71^ and would be consistent with HEY2 playing a role in other cell types apart from its characterized function in cardiomyocytes^70^. Similarly, the models for T cells and mast cells all captured an inverse relationship between expression and SMAD4 (Fig. 5e). TGFB signaling via nuclear translocation of SMAD4 is highly cell-type-specific, driving a broadly immune-suppressive role^72^ with SMAD4 alternately acting as a transcriptional repressor or activator depending on cellular context^73^. In addition, we observed cases where a particular TF motif was utilized by models across cell types, such as ZNF384 and YY1 motifs leading to increased expression predictions in all cell types (Fig. 5d). Such relationships are difficult if not impossible to detect in a testing strategy looking for enrichments in a cell type compared to others. In summary, predictive models identify TFs that play cell-type-specific roles, TFs related to expression across many cell types, and assign an explicit direction of effect on transcription.

## Discussion

We generated a resource of snATAC- and snRNA-Seq from multiple donors and utilized a state-of-the-art regression approach to find alterations by sex and age. Incorporating external snRNA-Seq datasets, we then studied sex- and age-dependent variation in a larger cohort, finding changes in cell-type proportions and transcriptional programs. We additionally studied the regulatory roles of transcription factors in distinct cell populations through enrichment analyses and predictive expression models. Performing single-cell or single-nucleus RNA analysis in solid human tissue is difficult, and our dataset represents one of only a small handful of studies covering the healthy human heart at single-cell resolution from multiple donors^6–8,17^.

Analysis of our newly generated data identified multimodal variation, such as transcriptional variation in metabolic pathways by sex (Fig. 2b) accompanied by altered accessibility for DNA binding motifs of glucose regulators (Fig. 2c). Analysis of six snRNA-Seq datasets identified variation in cell-type proportions and transcriptomes of cardiomyocytes by sex (Fig. 3e, g, j) and neurons by age (Fig. 3h, i). Our combined analysis also detected numerous pathways whose components show transcriptional variation as a function of age and sex across cell types. We see metabolic changes as a function of both covariates and multiple elements of immune activation with age (Fig. 3e, f), recapitulating changes we observed in our newly generated snRNA-Seq (Fig. 2b, d) and snATAC-Seq data (Fig. 2c, e). In both single-study and combined analyses, our findings point to the value of single-cell data in studying inter-individual variation, in contrast to the recent analysis of bulk RNA-Seq^74^. For example, we find expression levels of adipogenesis- and oxidative phosphorylation-related transcripts to be elevated in female cardiomyocytes versus male donors, but those same pathways have higher expression in males in lymphocytes (Fig. 3e).

We note some discrepancies between data modalities. For instance, analysis of our newly generated RNA-Seq dataset identified alterations in TGFβ signaling- and EMT-associated transcripts in macrophages by sex (Fig. 2b) but in our ATAC-Seq analyses we did not find significant alterations in motifs for SMAD3 – a canonical mediator of TGFβ signaling – in macrophages by sex (Fig. 2c). Sample size and inherently noisy single-nucleus data may underpin such discrepancies observed, like this distinction between sex-specific signatures in macrophages in RNA-Seq versus ATAC-Seq analyses. Furthermore, we note a handful of disagreements between analyses of newly generated data and combined datasets. For example, in our dataset, we see signatures of TGFβ signaling in females in RNA-Seq (Fig. 2b) and altered accessibility of canonical TGFβ signaling-associated motifs by sex in ATAC-Seq data (Fig. 2c), while in our meta-analysis we find no sex-specific changes in TGFβ hallmarks and elevated EMT hallmarks (a potential effect of TGFβ signaling) in males, not females (Fig. 3e). Given intrinsic variability, some differences may represent the larger analysis correcting spurious findings of the smaller. However, meta-analyses themselves present challenges, as differences in sample handling, sequencing platforms, and bioinformatic processing may contribute to differences between the datasets. Even in our larger analyses, sample size, and limited patient metadata preclude thoroughly controlling for clinical history, limit studying fine-grained changes like sex-specific differences before or after menopause onset^75^, and prevent the use of more flexible modeling approaches that could account for non-linear effects or covariate interactions. Fundamentally, the use of observational data also presents limitations in controlling for covariate effects. For example, a given illness could both act as a confounder and a consequence of aging-related changes. Finally, analyses of new datasets will be required to study differences occurring in cell populations like atrial cardiomyocytes^6,7^ that we did not study because of limited anatomical coverage.

In coming years, the field will find value from the generation of considerably larger amounts of data. Broad pathway-level concordance between analysis of newly generated data alone (Fig. 2d) and in conjunction with additional datasets (Fig. 3f) in detecting transcriptional changes like altered metabolism and immune signaling by age bodes well for the ability to incorporate datasets across platforms and sources to detect cell-type-specific changes. To find generalizable alterations in cell-type composition, careful and standardized tissue collection protocols will be key in controlling for confounding effects of inter-anatomical site variation. Greater availability of donor health and environmental history represents a particularly crucial area of attention as datasets proliferate, given the scope of human variation and the modest information typically available^6,7,15,36,37^. Additionally, perturbational work will be crucial for overcoming limitations inherent to observational designs and understanding the nature of observed alterations. For instance, reproducible findings of varied TGFβ signatures with age would immediately raise questions over whether such signatures are due to canonical TGFβ signaling or crosstalk with alternate signaling pathways. Our work and related studies inherently represent large-scale hypothesis refinement, identifying correlations between patient covariates and molecular signatures. For now, our identification of biological processes such as metabolic shifts, TGFβ signaling, and inflammation raises the prospect of alterations by sex or age that are already of clinical interest in cardiac disease^76–78^.

Comparison of TF motif presence in accessible chromatin of fetal cells versus adult cells indicated broadly correlated enrichments or depletion, with a handful of outliers representing putative developmental-stage-specific factors (Fig. 4). Experimental validation of such stage-specific activity will be challenging given the need to study and perturb parallel adult and fetal model systems. Despite the difficulty, understanding stage-specific regulatory roles could be valuable in explaining the onset of developmental disorders. For example, mutations in adult-specific regulatory factors manifest in heart disease only during childhood onward, rather than during embryonic stages.

We additionally quantified the extent of transcriptional variation that can be accounted for using a simple, binary TF motif-based linear model (Fig. 5b). Our results underscore the importance of distal regulatory information^66,69,79^ and reaffirm that simple models using TF motifs explain a minority of variation in RNA levels^64,65^. Furthermore, we identify regulatory factors in a method that complements widely-used tests^8,17,23^ based on motif presence in accessible DNA (Fig. 1f). In future work, complex models like deep neural networks^20–22^ will be required to fully leverage the information in distal DNA sequence. It will be particularly interesting to test TF roles found via our models (Fig. 5d), given the inherent co-occurrence of motifs in the genome (Supplementary Fig. 5d) and the risk that regularized linear models such as ours may utilize information from only a subset of motifs from a “family” of related features.

## Methods

### Tissue collection

This study complies with all relevant ethical regulations and was approved by the University of Washington Institutional Review Board (STUDY00002144). Informed consent was obtained prior to the collection of human tissues. No compensation was provided for participation. Collected samples were absent of evidence of disease upon review by study clinicians. Details regarding the collection are available on protocols.io^80,81^.

### Single-nucleus library generation

Nuclei for sci RNA-Seq were extracted from frozen, powdered heart tissue. 200–250 mg of frozen tissue was powdered while frozen, then dissociated using a Gentle MACS Tissue Dissociator at 4 °C using 5 mL of ice-cold lysis/fixation buffer containing 10 mM sodium phosphate (pH 7.2), 3 mM MgCl_2_, 10 mM NaCl, .02% Triton X-100, 5% glutaraldehyde, 1% DEPC, 10 mM ribonucleoside vanadyl complex (NEB). Dissociated tissue was filtered through a 70 µM cell strainer on ice and washed with an additional 5 mL of ice-cold lysis/fixation buffer. The buffer/nuclei mixture was then incubated for 15 min at 4 °C in a rotating 15 mL Falcon Tube. Nuclei were pelleted by centrifugation at 600 RCF for 8 min at 4 °C. Supernatant was decanted, then nuclei were resuspended in 1 mL of nuclei suspension buffer (NSB) containing 10 mM Tris HCl, pH 7.4. 10 mM NaCl, 3 mM MgCl_2_, 1% SuperaseIn, 1% bovine serum albumin (BSA) solution (NEB, 20 mg/mL). Nuclei were pelleted at 600 RCF for 5 min at 4 °C, and the supernatant was decanted. Nuclei were resuspended in 100 µL of NSB per aliquot, then snap-frozen with liquid nitrogen.

Libraries were generated using a 3-level sci RNA-Seq workflow ^24^. The workflow was modified to add a FACS sorting step following ligation in order to minimize background RNA levels, with a detailed workflow available at protocols.io (https://www.protocols.io/view/3-level-sci-rna-seq-with-facs-dm6gpw255lzp/v1). Libraries were sequenced using an Illumina Nextseq 500 high-output sequencing kit.

Nuclei for sci ATAC-Seq were extracted from powdered, frozen tissue and fixed as in previous work^23^. Libraries were generated using a 3-level sci ATAC-Seq workflow^23^ and sequenced using an Illumina Novaseq sequencer.

### Single-nucleus RNA-Seq analysis of newly generated data

Raw sequencing output was processed using a pair of Nextflow processing pipelines available at https://github.com/bbi-lab/bbi-dmux (handling sample demultiplexing) and https://github.com/bbi-lab/bbi-sci (handling assignments of reads to cells, filtering, alignment, and cell-by-gene matrix generation).

Analysis of single-cell RNA-Seq data was performed using Monocle 3^24^. Cells were filtered by discarding any with unique molecular identifiers (UMIs) less than 100, mitochondrial RNA percentage greater than 10, or a Scrublet doublet score^82^ of over 0.2. A 2-dimensional UMAP representation^83^ of cells was found after using mutual nearest neighbors alignment^84^ to align by sample. Cell-type assignments were made manually based on the expression of marker genes in UMAP clusters (Fig. 1d). Plots of several QC metrics - overlaid over 2D UMAP embeddings or shown as distributions per sample - are available in Supplementary Fig. 1.

### Datasets for meta-analysis of age- and sex-dependent variation

Five additional datasets were combined with our newly generated data for a combined dataset drawing from 73 unique donors (Supplementary Data 15 and 16). In publications where both single-cell and single-nucleus data was generated, we only utilized single-nucleus RNA-Seq data given qualitatively large expression alterations as a function of nucleus vs. cell data (Supplemental Fig. 6) and the larger number of single-nucleus datasets available. We used cell-type assignments for individual transcriptomes as assigned by original publications, harmonizing distinct naming conventions as described in Supplementary Data 7. Donor sex and age were obtained from publication metadata, and in cases where only age ranges were given, we used the median of a given age range as the donor’s age for use in our regression analyses (e.g. a range of 60–65 was converted to 62.5 years).

UMAP visualizations of combined datasets used mutual nearest neighbor alignment to correct for variation by publication (Fig. 3b, c) and down-sampled nuclei 5-fold randomly (only for these visualizations, not in any downstream analysis) to speed computation.

### Single-nucleus ATAC-Seq analysis

Sequencing output was processed using a pair of Nextflow processing pipelines available at https://github.com/bbi-lab/bbi-sciatac-demux (handling demultiplexing) and https://github.com/bbi-lab/bbi-sciatac-analyze (assigning reads to cells, aligning reads, calculating peaks, finding motif occurrences in peaks, and generating cell x peak matrices).

Analysis of single-cell ATAC-Seq data was performed using Monocle 3^24^. Cells were filtered by discarding any with unique molecular identifiers (UMIs) less than 1000, fractions of reads in TSS (FRIT) of less than 0.08, fractions of reads in peaks (FRIP) less than 0.2, or a doublet likelihood of greater than 0.5^82^. Gene activity scores were calculated using ArchR^85^ using default settings. Cell-by-gene activity score matrices were then used to generate a Monocle CDS object. The ATAC-Seq data was then aligned with the filtered RNA-Seq data using Harmony^86^ based on all genes shared between RNA and ATAC CDS objects, and a new UMAP embedding was generated based on the corrected PCA coordinates of both datasets after Harmony correction. Based on UMAP coordinates in this new embedding, ATAC-Seq cells were labeled using a k-nearest neighbor transfer from the *k* = 7 nearest RNA-Seq cells (using cell assignments described above for RNA-Seq data). Plots of several QC metrics - overlaid over 2D UMAP embeddings or shown as distributions per sample - are available in Supplementary Fig. 2.

### Differential motif abundance testing in accessible peaks

The presence of TF motifs in peaks was calculated based on the presence of any motif occurrence in the peak DNA sequence below a *p*-value cutoff of 1e−7 using MOODS^87^. Motif-count × cell matrices were then made by multiplying a motif (rows) × peaks (columns) matrix with a peak (rows) × cell (columns) matrix, generating a motif-count × cell matrix where each entry corresponded to the number of peaks accessible in a given cell that contained a given TF motif.

To test for motif abundances that varied by a function of donor covariates (age and sex), testing was run separately for all cells of a single cell type. Testing for motif counts was done using a generalized linear mixed model fit using the lme4 package^88^, using a negative binomial model with sample donor as a random effect, as well as fixed effects of anatomical site, donor age, and sex. *P*-values were calculated using a *z*-test from fit coefficient estimates and standard errors, then multiple-testing correction was performed with the Benjamini–Hochberg procedure^89^. Enrichments that were statistically significant at FDR < 0.1 were shown (Fig. 1f). This modeling approach is available in current releases of Monocle 3^24^.

For testing of cell-type-specific motif enrichments (Fig. 1f), testing was run for all cells at once. To test for motifs enriched in a specific cell type, all cells were assigned a dummy variable valued as ‘1’ for cells that are from the type being tested, and ‘0’ for all others. Testing was then run using a GLMM fit using the lme4 package^88^, using a negative binomial model with sample donors as a random effect, as well as fixed effects of the cell-type-dummy variable, anatomical site, donor age, and sex. *P*-values were calculated using a *z*-test from fit coefficient estimates and standard errors, then multiple-testing correction was performed with the Benjamini–Hochberg procedure^89^.

### Differential expression testing and pathway analysis

DE testing for newly generated datasets (Fig. 2a, b, d) used a generalized linear mixed model fit using the lme4 package^88^, using a negative binomial model with sample donor as a random effect, as well as fixed effects of anatomical site, donor age, and donor sex. This modeling approach is available in current releases of Monocle 3^24^. *P*-values for fixed effect coefficients were obtained using a *z*-test based on coefficient estimates and standard errors of those estimates, then *q-*values were derived from those *p*-values using the Benjamini–Hochberg procedure^89^ for multiple-testing correction.

For DE testing in our meta-analysis of multiple datasets, we used the Nebula package^90^ to fit a GLMM in a feasible amount of time on the much larger number of cells involved. We modeled donor-specific variation as a random effect while modeling data source, age, sex, and log10(UMI), and anatomical site of cells as fixed effects. We used the default random effect structure of the Nebula package, an approximated form of a negative binomial gamma mixed model^90^. As in the above fitting method, *p*-values for fixed effect coefficients were obtained using a *z-*test based on coefficient estimates and standard errors of those estimates, then *q*-values were derived from those *p*-values using the Benjamini–Hochberg procedure^89^ for multiple-testing correction. For anatomical site fixed effects, the left ventricle (LV) was used as the baseline level. Thus, the fit model explicitly finds effects for the septum, apex, and right ventricle (RV) with respect to LV. These tests in reference to LV expression levels are reported in Supplementary Data 8–10 and shown in Supplementary Fig. 3d–g.

Gene set enrichment analysis tested for enrichments by age or sex within 50 Hallmark Pathways accessed from the MSigDB collection^91^ accessed through the msigdbr R package. Testing used the fgsea package^92^ to find gene set enrichments in these pathways as a function of age or sex based on the test statistic (coefficient / standard error) fit using the mixed model described above for each covariate. *P*-values are generated based on permutation tests seeing if the genes belonging to a given pathway are overall higher or lower than expected by chance in a list of all genes ordered by their test (*z*) statistic. Next, multiple-testing correction was performed with the Benjamini–Hochberg procedure^89^ to generate *q*-values for each pathway.

### Testing for variation in cell-type proportions

We tested for alterations in cell-type composition as a function of age or sex using a beta-binomial mixed effect model^93^. In both analyses of newly generated data (Supplemental Data 6) and meta-analysis (Fig. 3g, h) we capture variation by sex, age, and anatomical site as fixed effects while modeling donor as a random effect. In the meta-analysis (Fig. 3g, h) we include an additional fixed effect term to account for variation by data source and the inherent correlation between different samples (representing distinct anatomical sites) derived from a single donor. Models were fit using the default parameters in ProReg^93^, calculating *p*-values based on a *z*-test with respect to fixed effect coefficients and standard errors, then calculating *q*-values by applying the Benjamini–Hochberg procedure^89^.

In these analyses, the independent unit of measurement is unique donors: *n* = 8 in analyses of new data, *n* = 73 in the meta-analysis. The model uses more individual data points than this (14 in the analysis of new data, 128 in the meta-analysis) with some cases of individual donors having multiple samples taken from distinct anatomical sites. The use of a mixed effect model with a random effect by donor accounts for this distinction between the total number of data points and the true “*n*” of independent sources of data through modeling donor identity as a random effect during model fitting, capturing the inherent correlation between points drawn from a single donor.

In the plot of neuron abundance by age (Fig. 3h) the curve is based on the predicted neuron proportion of a sample by age given the age coefficient and intercept obtained by fitting a mixed effect beta-binomial model for neuron proportions. The error range represents the ranges between curves for predicted proportions if the age coefficient were ± 2 standard errors from the actual estimated coefficient. For individual points shown, proportions were normalized by regressing out the effect of sex, anatomical site, and study on cell proportion based on coefficients for those covariates obtained by fitting a mixed effect beta-binomial model. Thus each point represents the best estimate for what the measured neuron proportion in a given sample would be if there were not systematic increases or decreases across studies, by sample site, or between the sexes. For example, if the model fits a coefficient of “1.5” for male samples, normalized neuron proportions in a male would be adjusted to be

1 + (1 + logit(measured_proportion) + 1.5 * is_male)^−^^1^

as the beta-binomial model uses a logit link function.

### Adult versus fetal enrichment comparisons

Enrichments for TF motifs in accessible chromatin of fetal cell types was accessed at https://descartes.brotmanbaty.org/bbi/human-chromatin-during-development/ (see “Motif enrichment across cell types” section for download link). Enrichments in adult cell types were calculated as described above under “Differential motif abundance testing in accessible peaks”. Comparisons were made between the following adult-to-fetal matchings: “Cardiomyocyte” and “Cardiomyocytes”; “Vascular Endothelium” and “Vascular endothelial cells”; “Endocardium” and “Endocardial cells”; “Macrophage” and “Myeloid cells”; “Perivascular Cells” and “Smooth muscle cells”; “Fibroblasts” and “Stromal cells”; “Adipocytes” and “Epicardial fat cells”; “Neuronal” and “Purkinje neurons”; “T Cells” and “Thymocytes”.

For each comparison, plots (Fig. 4a–d**;** Supplementary Fig. 4**;** Supplementary Data 14) were calculated using only motifs that were significantly enriched in either adult or fetal data at an FDR cutoff of 0.1. Adult enrichments and corresponding *q*-values were found as described in “Differential motif abundance testing in accessible peaks” while fetal enrichment statistics were taken from^23^. Outliers were selected based on qualitative divergence from broad cell-type correlations in enrichments between fetal and adult cells.

### RNA expression predictive modeling

First, pseudo-bulk expression levels were calculated by pooling all UMIs for all genes for cells within a particular cell type. These were used to quantify the transcripts per million for each gene. Log2(TPM) was then used as the RNA expression level to be predicted for a particular gene/cell-type pair.

To link distal sites to promoters, we ran Cicero^69^ to quantify covariance among peaks across all cell types. To link distal sites to genes, we first defined any peaks that intersected a defined window around the TSS (this region size was a hyperparameter set through performance on a validation set, see below). Then, any peaks outside the promoter set of peaks that were linked with a co-accessibility score greater than some cutoff (a hyperparameter). Motifs from the JASPAR database^94^ “2018 Non-redundant Vertebrates” motif set were determined using FIMO^95^ at varying *p*-value cutoffs (varied as a hyperparameter). For models using promoter sequence only, features would be a binary value for if one or more motif occurrences was found in the promoter sequence below a *p*-value cutoff. For models using promoter and distal sequence, features were binary values for if a motif occurred in the promoter or distal regions.

RNA expression for protein-coding genes was predicted with an elastic net linear model using motif presence/absence as features. Data was divided into train, test, and validation sets at the level of chromosomes (approximately an 80/10/10 split in gene numbers) for a hyperparameter setting. First, promoter size and motif *p*-values were varied (Promoter sizes upstream/downstream of TSS were 1000/200, 1500/500, and 5000/2000. *P*-values tested were 1e−4, 1e−5, 1e−6). Models were trained on the training set setting l1/l2 penalties by internal cross-validation, and then evaluated on the validation set. The best average performance occurred using a *p*-value cutoff of 1e−4 with a promoter of 1500/500 bases upstream/downstream. Holding that promoter region size constant, we trained models varying the cicero co-accessibility cutoff to link a distal site, the maximum number of distal sites to link, and the window size of DNA bases to scan centered at a linked peak. We tested combinations of Co-accessibility cutoffs of 0.015, 0.035, 0.05; max distal sites of 5, 10, or 20; distal sit size of 600 or 1,000 bases, motif *p*-value cutoffs of 1e−4, 1e−5, and 1e−6. Models were trained on the training set setting l1/l2 penalties by internal cross-validation, then evaluated on the validation set (Supplementary Fig. 5A). Optimal performance was obtained using a co-accessibility cutoff of .015, a maximum of 20 distal sites, 1000 bp distal site windows, and a motif cutoff of 1e−4. Those parameters were then set for use in training a model for evaluation on the test set. L1/l2 penalties were set by internal cross-validation on a pooled training + validation set, and then a model was trained using those penalties and the best hyperparameters found earlier. The model was then evaluated on the test set (Fig. 5b). Finally, holding those hyperparameters constant, a final model was trained using all three train, validation, and test sets. The coefficients of this final fit model are reported in Fig. 5d.

### Supplementary information

Supplementary Information

Description of Additional Supplementary Files

Supplementary Data 1

Supplementary Data 2

Supplementary Data 3

Supplementary Data 4

Supplementary Data 5

Supplementary Data 6

Supplementary Data 7

Supplementary Data 8

Supplementary Data 9

Supplementary Data 10

Supplementary Data 11

Supplementary Data 12

Supplementary Data 13

Supplementary Data 14

Supplementary Data 15

Supplementary Data 16

Supplementary Data 17

## Data Availability

Raw data is available through the HuBMAP consortium website’s data portal (https://portal.hubmapconsortium.org/). Dataset IDs for samples presented in this study are available in Supplementary Data 17. Processed files underpinning all analysis in the manuscript are available on AWS S3 storage (http://trapnell-lab-s3-heart-sc-dr.s3-website-us-west-2.amazonaws.com/) including Monocle CDS objects for all single-cell data presented.

## Code availability

Code is accessible in a GitHub repository at https://github.com/Daread/hubmapWork. Work used Monocle version 3_0.2.3.0, ArchR version 1.0.1, Seurat version 3.2.0. Code as it existed at the time of publication is available at 10.5281/zenodo.12682674^96^.
