# Supplementary material for: Single-cell analysis of chromatin and expression reveals age- and sex-associated alterations in the human heart
Source: Commun Biol. 2024 Aug 26;7:1052. doi: 10.1038/s42003-024-06582-y (PMC11347658; doi:10.1038/s42003-024-06582-y)
Supplement: Supplementary file 1 — Supplementary Information [file 42003_2024_6582_MOESM1_ESM.pdf]

Supplementary Figure 1

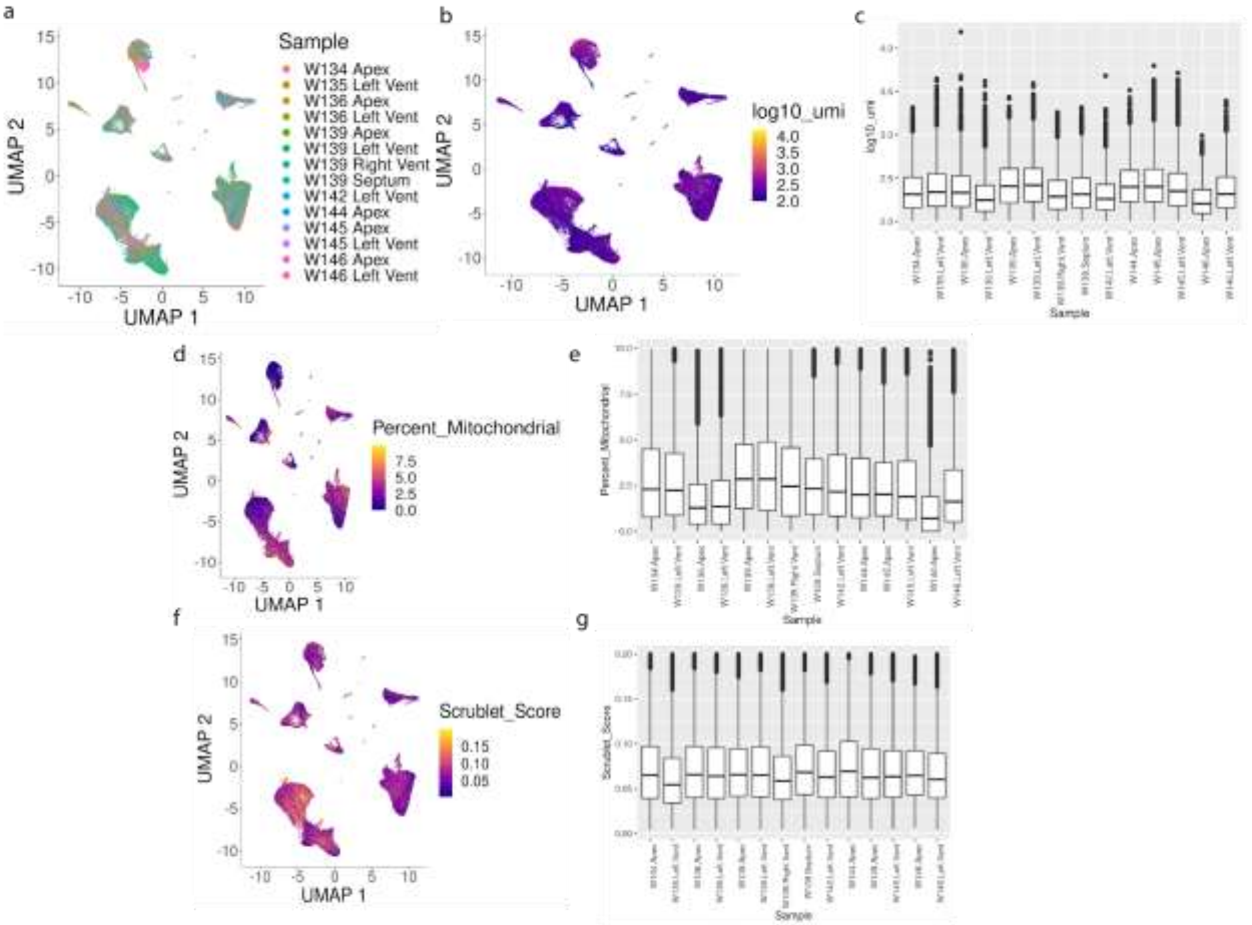

Supplemental Figure 1: QC metrics for sci RNA-Seq. A) UMAP embedding of all sci RNA-Seq data coloring cells by sample of origin. B) UMAP coloring by log10(unique molecular identifiers per cell). C) Boxplot showing log10(UMI) by sample. D) UMAP coloring by percent of UMIs coming from mitochondrial RNA. E) Boxplot showing the percent of UMIs from mitochondrial RNA, by sample. F) UMAP coloring by the score (0 to 1 range) given by Scrublet, assessing likeness of a cell's transcriptome to a simulated distribution of doublets. G) Boxplot showing scrublet scores by sample.

Supplementary Figure 2

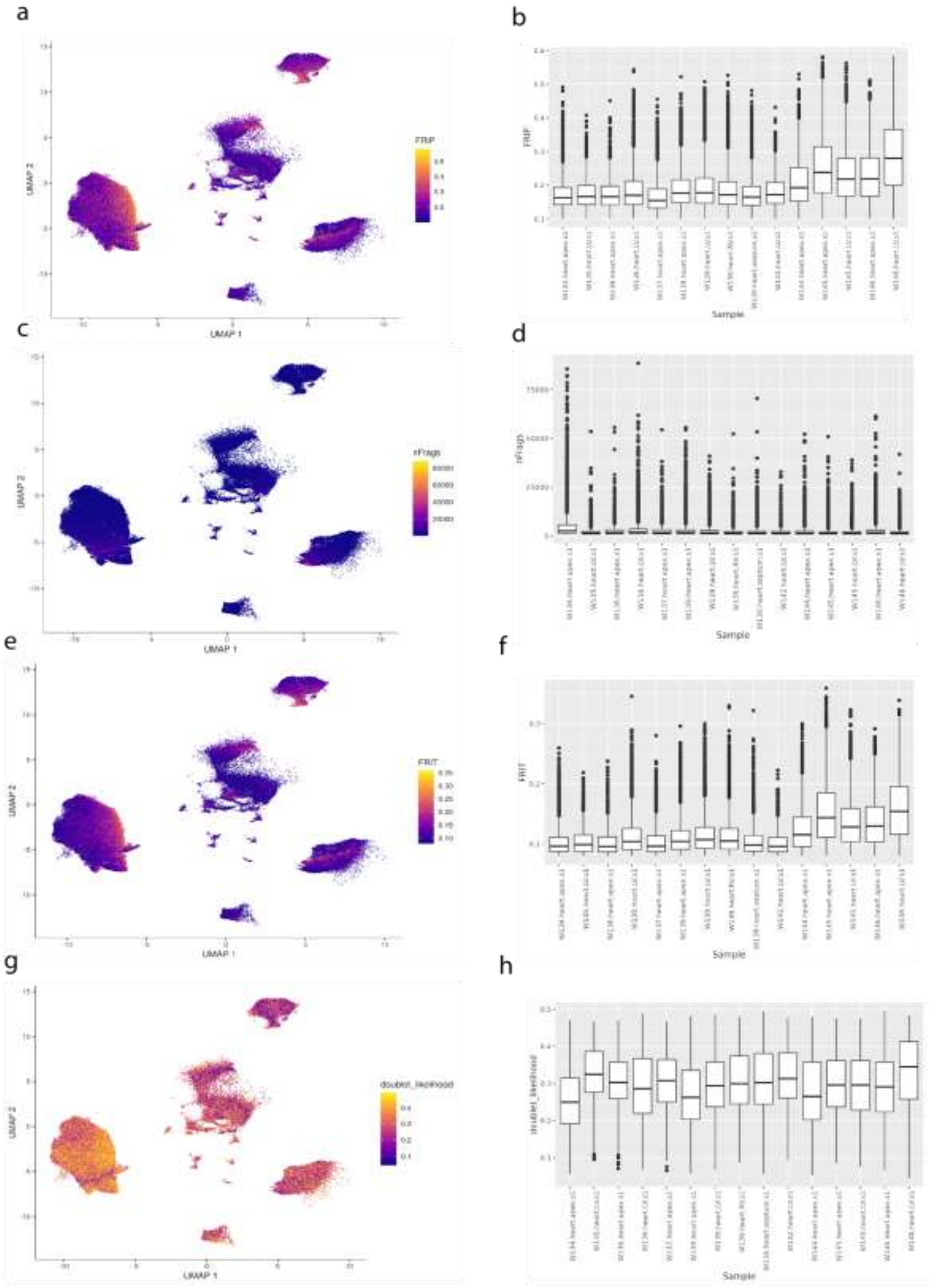

Supplemental Figure 2: QC metrics for sci ATAC-Seq. A) UMAP embedding of ATAC-Seq cells (after co-embedding with RNA data, cells from RNA-Seq not shown) coloring by fraction of reads in peaks (FRIP). B) FRIP distribution by cell, split by sample. C) UMAP embedding of ATAC-Seq profiles coloring by number of unique fragments captured (nFrags) per cell D) Distribution of fragments captured per cell, split by sample. E) UMAP embedding of ATAC-Seq profiles coloring by fraction of reads in TSS regions (FRIT) per cell F) Distribution of FRIT scores per cell, split by sample G) UMAP embedding of ATAC-Seq profiles coloring by the doublet likelihood assigned to each cell based on similarity to in-silico doublets. H) Distribution of doublet likelihood scores by celll, split by sample.

Supplementary Figure 3:

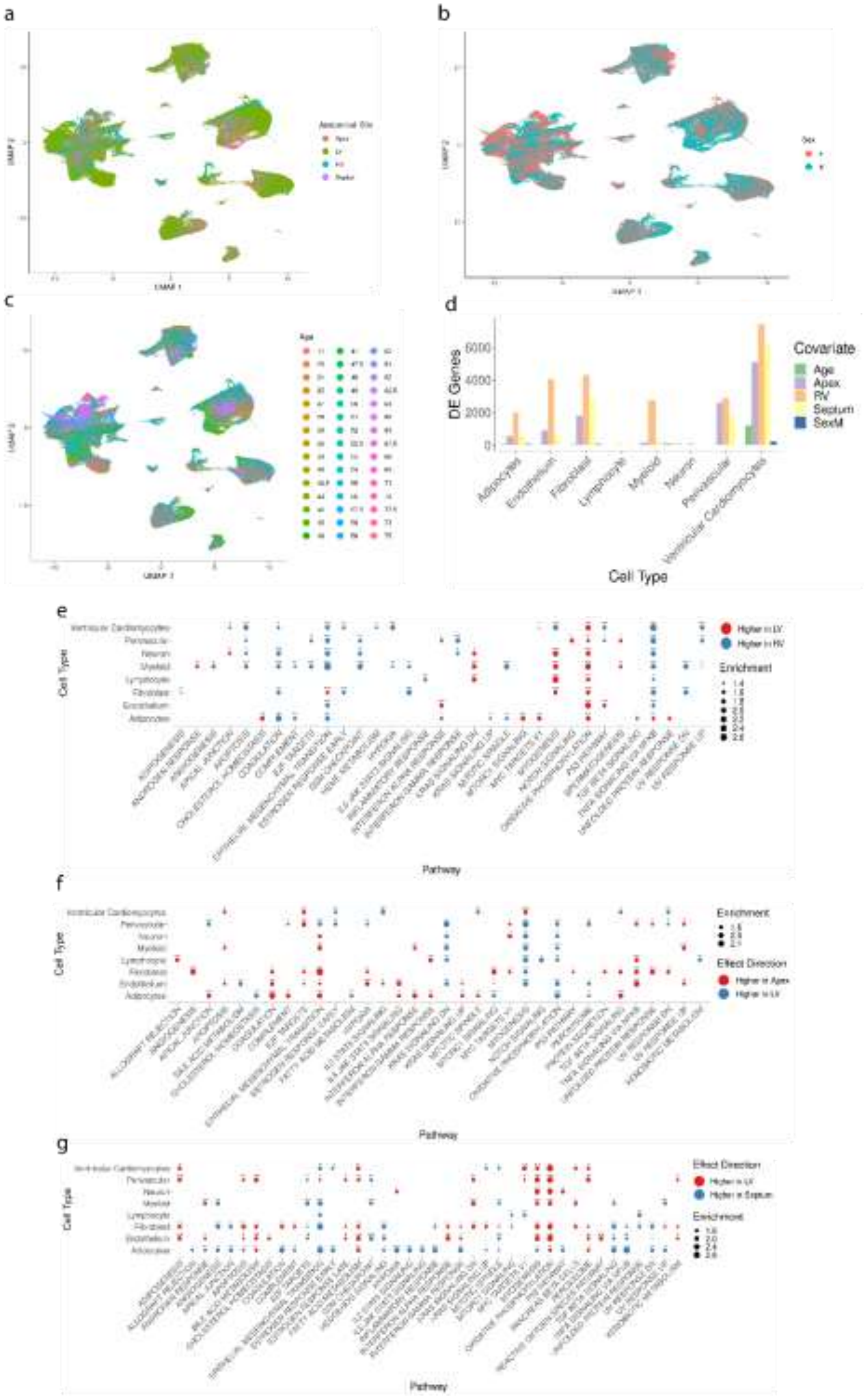

Supplementary Figure 3: UMAP embedding of datasets used in combined analysis of variation by age and sex. One point represents on single transcriptome, points are colored by A) anatomical site, B) sex of donor, or C) age of donor. D) Number of differentially expressed genes as a function of age, sex, or anatomical site with respect to left ventricle (LV) as the reference level for apex, right ventricle (RV), or septum. FDR = 0.1. Testing used a mixed effect model with fixed effects for sex, age, anatomical site, cell UMI, data source, with a random effect for donor identity (see methods). Uses n=73 donors. E) GSEA by biological pathways as a function of an LV vs. RV comparison, looking at variation as fit using a mixed effect model (see methods). Red = pathway up-regulated in left ventricle (LV), Blue = up-regulated in right venticle (RV). Total donors n = 73. Asterisk number indicates FDR threshold of significance. Benjamini Hochberg method for multiple testing correction. F) GSEA as in panel E, but enrichments between apex and left ventricle. G) GSEA as in panel E, but testing enrichments between septum and left ventricle.

Supplementary Figure 4:

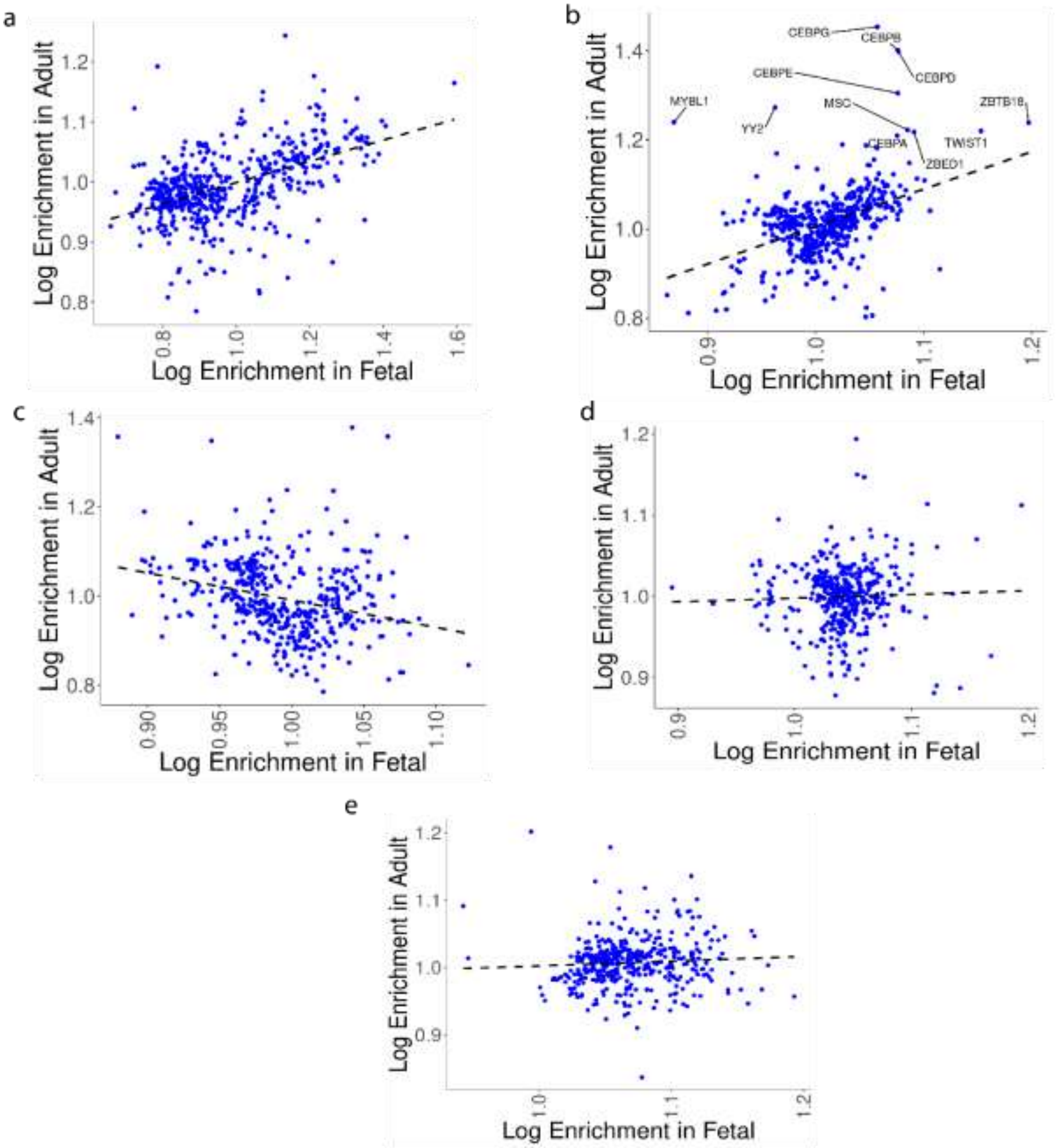

Supplemental Figure 4: Enrichment of TF motifs in the accessible peaks of fetal or adult sn ATAC-Seq. A) Enrichments in T Cells (Adult) versus thymocytes (fetal). Enrichments are shown for TFs that were statistically enriched in one or both of fetal or adult analyses (FDR = .1). B) Enrichments in fibroblasts (adult) versus stromal cells (fetal). C) Enrichments in endocardial cells. D) Enrichments in perivascular cells (adult) versus smooth muscle cells (fetal). E) Enrichments in adipocytes.

Supplementary Figure 5:

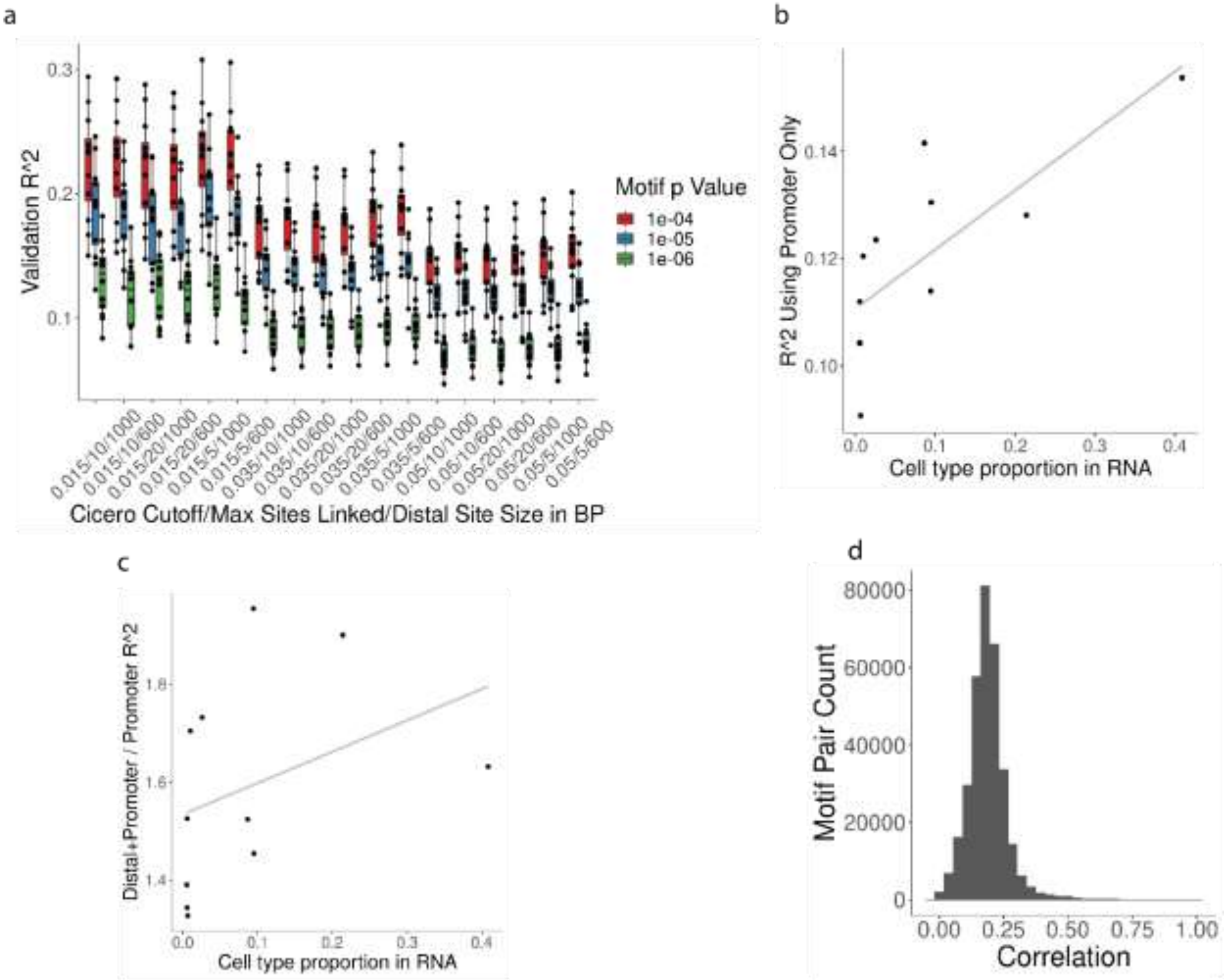

Supplemental Figure 5: Predictive models of RNA expression. A) R^2 for cell type-specific models (one point = one cell type) using varied p value cutoffs for calling motif presence in FIMO (color) and varied co-accessiblity scores for linking distal sites to promoters ("Cicero Cutoff"), varying maximum number of distal sites to be linked to a promoter ("Max Sites Linked"), and varying sizes of distal sites in base pairs ("Distal Site Size in BP"). B) Accuracy of cell type-specific models using only motifs found in promoter regions (y-axis) compared to the proportion of that cell type in RNA-Seq data (x-axis). C) The ratio of a distal+promoter / promoter-only models, for each cell type (y-axis) compared to the proportion of that cell type in RNA-Seq data. D) Distribution of correlations between motifs across all protien coding genes within the dataset used for final model fitting (e.g. using the same hyperparameters as were used in the models evaluated in Fig. 5B).

Supplementary Figure 6:

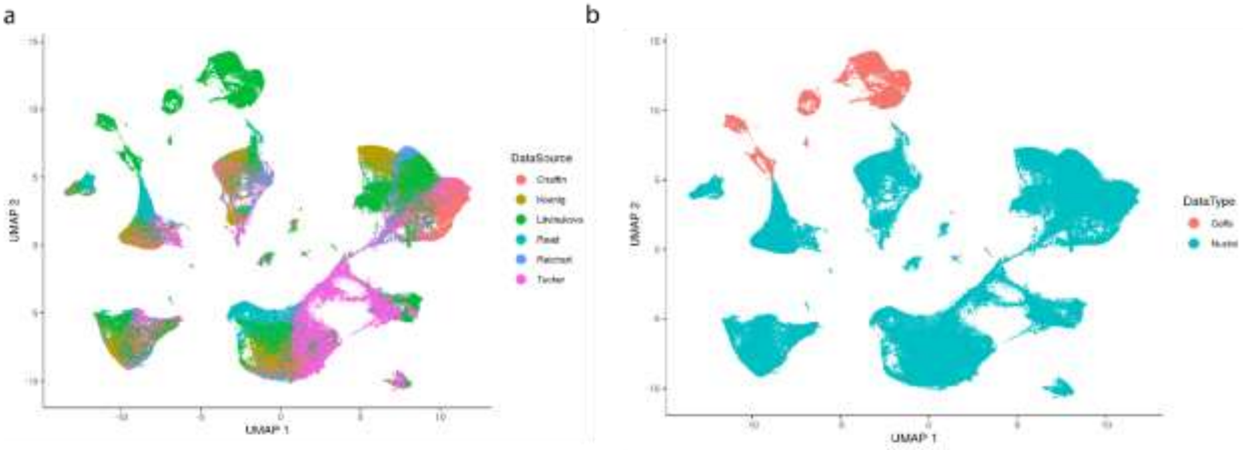

Supplementary Figure 6: A) UMAP embedding of datasets used in combined analysis of variation by age and sex. One point represents on single transcriptome, points are colored by study of origin. B) The same UMAP embedding, colored by whether the data was single-nucleus RNA-Seq (Blue) or single-cell RNA-Seq (Red).
