# Supplementary material for: Single-cell analysis of chromatin and expression reveals age- and sex-associated alterations in the human heart
Source: Commun Biol. 2024 Aug 26;7:1052. doi: 10.1038/s42003-024-06582-y (PMC11347658; doi:10.1038/s42003-024-06582-y)
Supplement: Supplementary file 2 — Description of Additional Supplementary Files [file 42003_2024_6582_MOESM2_ESM.pdf]

Description of Additional Supplementary Files

File name: Supplementary Data 1

Description: Table detailing tissue samples used for generation of snRNA- and ATAC-Seq data.

File name: Supplementary Data 2

Description: Differential expression results for tests by age, FDR = 0.1

File name: Supplementary Data 3

Description: Differential expression results for tests by sex, FDR = 0.1

File name: Supplementary Data 4

Description: Differential accessibility testing results for tests by sex, FDR = 0.1

File name: Supplementary Data 5

Description: Differential accessibility testing results for tests by age, FDR = 0.1

File name: Supplementary Data 6

Description: Results of beta-binomial regression tests for cell type proportions varying by age/sex.

File name: Supplementary Data 7

Description: Information summarizing samples/donors used in meta-analysis of snRNA-Seq

File name: Supplementary Data 8

Description: Differential expression results for tests of Septum vs. Left Ventricle from meta-analysis of snRNA-Seq

File name: Supplementary Data 9

Description: Differential expression results for tests of Apex vs. Left Ventricle from meta-analysis of snRNA-Seq

File name: Supplementary Data 10

Description: Differential expression results for tests of Right Ventricle vs. Left Ventricle from meta-analysis of snRNA-Seq

File name: Supplementary Data 11

Description: Differential expression results for variation by age from meta-analysis of snRNA-Seq

File name: Supplementary Data 12

Description: Differential expression results for variation by sex from meta-analysis of snRNA-Seq

File name: Supplementary Data 13

Description: Tests results for regression testing of cell type proportions in meta-analysis of snRNA-Seq

File name: Supplementary Data 14

Description: Correlations across cell-type enrichments in accessible chromatin for all motifs tested for comparisons between corresponding adult and fetal cell types.

File name: Supplementary Data 15

Description: Table summarizing harmonization of naming conventions for cell types used in meta-analysis of snRNA-Seq.

File name: Supplementary Data 16

Description: Table detailing donor information for tissue samples used in meta-analysis of snRNA-Seq.

File name: Supplementary Data 17

Description: Information for identifiers to access data corresponding to samples presented/analyzed in this manuscript on the HuBMAP online portal.
